# A Mouse Model for Parkinson Disease

**DOI:** 10.1371/journal.pbio.0030303

**Published:** 2005-08-02

The debilitating effects of Parkinson disease are well known: muscle rigidity, impaired movement, and the uncontrollable shaking that makes even the most mundane activity a challenge. The symptoms result from a progressive deterioration of the nervous system and degeneration of neurons that control motor function and coordinated movement. These neurons, found in the midbrain, produce dopamine, a chemical that transmits brain signals (called a neurotransmitter) that regulate motor control, energy levels, and motivation to achieve goals. In Parkinson disease, dopamine levels drop as neurons degenerate, producing the characteristic symptoms. With no cure on the horizon, the most common treatment involves administration of the dopamine precursor, L-DOPA, usually in pill form. Though symptoms subside at first, this treatment is rendered ineffective over time.

To develop better therapies, scientists use animal models of human diseases to test the efficacy and toxicity of potential treatments before moving on to human trials. To be effective, animal models must recapitulate the disease symptoms as closely as possible. However, while previous models of Parkinson with incomplete dopamine depletion captured some of the manifestations of this condition, the dominance of the dopamine system on the control of locomotion has made it difficult to elucidate the contribution of other neurotransmitter systems. In a new study, Tatyana Sotnikova and colleagues from Duke University created such a model that recapitulates many of the symptoms of Parkinson. By eliminating the dopamine transporter—the protein responsible for recycling the chemical into neurons—in mice, the authors reduced dopamine levels in the midbrain by 20-fold. In addition, chemically inhibiting dopamine production in these mice resulted in essentially unmeasurable levels of the neurotransmitter, since it could now neither be produced at normal levels nor be recycled. Because these mice exhibited the symptoms of Parkinson disease remarkably well, the authors could test how well drugs that act independently of dopamine ameliorated symptoms of the disease. This approach allows the identification of drugs that may serve to improve treatment at later stages of the disease, when dopamine-producing neurons have been severely reduced in number and L-DOPA efficacy has been reduced. [Fig pbio-0030303-g001]

**Figure pbio-0030303-g001:**
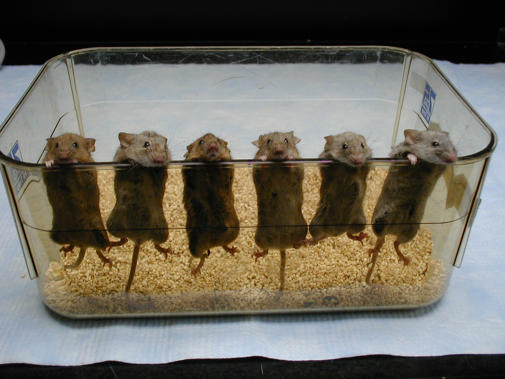
To model Parkinson disease, researchers bred mice with severe dopamine deficiencies that displayed rigidity, inhibited motion, and, as seen here, freezing behavior

The authors tested a number of drugs at various doses and found that in addition to L-DOPA-related treatments, drugs related to amphetamine were effective in ameliorating muscle rigidity, tremor, and impaired movement. Most effective was methylenedioxymethamphetamine HCl (MDMA), commonly known as ecstasy. It has been shown that amphetamines can trigger release of neurotransmitters such as dopamine, serotonin, and norepinephrine and cause sudden bursts in neurotransmission, leading to a feeling of alertness, increased muscular activity, and reduced fatigue. This study, however, shows that treating mice with MDMA does not increase dopamine levels; furthermore, treating the mice with drugs related to serotonin or norepinephrine did not ameliorate the disease's symptoms. These results suggest that MDMA likely acts through a pathway unrelated to these common neurotransmitters.

The authors tested the possibility that MDMA may be increasing transmission via receptors that respond to compounds that are normally present at very low levels, called trace amines. Activation of these receptors reduced rigidity and akinesia, as with MDMA, though to a much lower level. Thus, while it is possible that MDMA is acting through trace amine receptors, this may not be the only pathway used. Future studies will be required to elucidate how MDMA ameliorates Parkinson symptoms.

The largest effects of MDMA on reducing symptoms was seen at levels that produce neurotoxic effects in wild-type mice, though administering non-neurotoxic doses of MDMA along with tiny amounts of L-DOPA that are normally ineffective proved as effective as higher doses of MDMA alone. Interestingly, the authors report an absence of significant side effects of even very high doses of MDMA administration on mice lacking the dopamine transporter; however, since patients with Parkinson disease do not necessarily lack the dopamine transporter, toxicity of MDMA and related compounds will need to be studied in greater detail in the future. This study opens the door to a search for compounds related to ecstasy, which may provide a more effective treatment for symptoms of Parkinson in the later stages of the disease—and hopefully allow patients to perform the simple functions of everyday life independently again.

